# Relation of atrial electromechanical delay to P‐wave dispersion on surface ECG using vector velocity imaging in patients with hypertrophic cardiomyopathy

**DOI:** 10.1111/anec.12801

**Published:** 2020-09-24

**Authors:** Hala Mahfouz Badran, Ghada Soltan, Eslam Eltahan, Magdi H. Yacoub, Naglaa Faheem

**Affiliations:** ^1^ Cardiology Department Menofia University Shebin Elkom Egypt; ^2^ The BAHCM National Program Aswan Egypt; ^3^ Imperial College London UK

**Keywords:** atrial electromechanical functions, hypertrophic cardiomyopathy, left atrial electrical activity, vector velocity imaging

## Abstract

**Objectives:**

Heterogeneity of structural and electrophysiologic properties of atrial myocardium is common characteristic in hypertrophic cardiomyopathy (HCM). We assessed the dispersion of atrial refractoriness on surface ECG using P‐wave dispersion (PWD) and its relation to atrial electromechanical functions using vector velocity imaging (VVI) in HCM population.

**Methods:**

Seventy‐nine HCM patients (mean age: 43.7 ± 13 years, 67% male) were compared with 25 healthy individuals as control. P‐wave durations, P_max_ and P_min_, P‐wave dispersion (PWD), and P terminal force (PTF) were measured from 12‐lead ECG. LA segmental delay (TTP‐d) and dispersion (TTP‐SD) of electromechanical activation were derived from atrial strain rate curves.

**Results:**

HCM patients had longer PR interval, PW duration, higher PWD, PTF, QT_c_ compared to control (*p* < .001). HCM patients were classified according to presence of PWD into two groups, group I with PWD > 46 ms (*n* = 25) and group II PWD ≤ 46 ms (*n* = 54). Group I showed higher prevalence of female gender, higher PTF, QTc interval, left ventricular outflow tract (LVOT) obstruction, *p* < .01, LVOT gradient (*p* < .001), LV mass index (*p* < .01), E/E' (*p* < .01), and severe mitral regurgitation (*p* < .001). Moreover, PWD was associated with increased atrial electromechanical delay (TTP‐d) and LA mechanical dyssynchrony (TTP‐SD), *p* < .001. LA segmental delay and dispersion of electromechanical activation were distinctly higher among HCM patient.

**Conclusion:**

PWD is simple ECG criterion, and it is associated with more severe HCM phenotype and LA electromechanical delay while PTF is linked only to atrial remodeling.

## 1 INTRODUCTION

Hypertrophic cardiomyopathy (HCM) is a genetic heart disease with heterogeneous clinical expression, outcome, and management options (Maron et al., 2014; Maron et al., 2016). Left atrial (LA) remolding and dysfunction, (Maron et al., 2016) atrial fibrosis (Maron et al., 2014), and LA appendage dysfunction (Maron et al., 2015) have been described. They are recognized as common, important consequences (Maron et al., 2015) and known to lead to electro‐anatomical remodeling, alter the atrial conduction properties and act as a substrate for the occurrence of AF (Rowin et al., 2017).

P‐wave dispersion (PWD), defined as the difference between the maximum and minimum P‐wave duration on surface ECG, is a new electrocardiographic marker that has been associated with inhomogeneous and discontinuous propagation of sinus impulses (Magnani et al., 2010). The correlation between the presence of interatrial and intra‐atrial conduction abnormalities and the induction of paroxysmal atrial fibrillation (AF) has been well documented (Liu et al., 1998). The estimation of the probability of patients in developing paroxysmal AF might guide the clinician in the management and stratification of HCM patients at higher risk of developing AF.

Recently, strain and strain rate measured by novel vector velocity imaging (VVI) have been used in evaluating cardiac mechanics. It allows simultaneous and precise analysis of atrial mechanics during its different phases reservoir, conduit, and contractile functions (Badran et al., 2019; Pala et al., 2010; Sanders et al., 2003) in addition to measurement of atrial electromechanical delay (Acar et al., 2009; Rein et al., 2003).

Our hypothesis was that there might be a relationship between LA mechanical changes in HCM and the atrial electrophysiologic characteristics on the 12 lead ECG. In the present study, we investigated the relation of PWD on surface ECG to LA electromechanical functions using VVI in patients with HCM.

## 2 PATIENTS AND METHODS

### 2.1 Study population

Between January 2018 and May 2019, 79 consecutive HCM patients were referred to our echocardiographic laboratory for different purposes (diagnosis, risk stratification, regular follow‐up) and included in the study. They were examined in a single center (Yacoub Research Unit, Menoufia University, Egypt, and as a part of the BA‐HCM National Registry Program).

This is a cross‐sectional study in which patients with established diagnosis of HCM were included after their informed consent and after approval of ethical committee of Menoufia University. The diagnosis of HCM was based on echocardiographic demonstration of a nondilated, hypertrophic left ventricle (LV) with increased LV wall thickness (≥15) in one or more LV segments and in the absence of any identifiable causes capable of producing such hypertrophy (Maron et al., 2014, 2015, 2016). All patients were in sinus rhythm.

### 2.2 Exclusion criteria

Patients with AF, history of paroxysmal atrial fibrillation, coronary artery diseases, prior surgical myectomy, prior pacemaker, or implantable cardiovertor defibrillator (ICD) implantation, and patients with known phenocopies of HCM (e.g., Fabry disease, lysosomal associated membrane protein‐2 cardiomyopathy, or amyloidosis) or poor quality echocardiographic imaging were excluded from the study.

### 2.3 Electrocardiographic analysis

**FIGURE 1:**
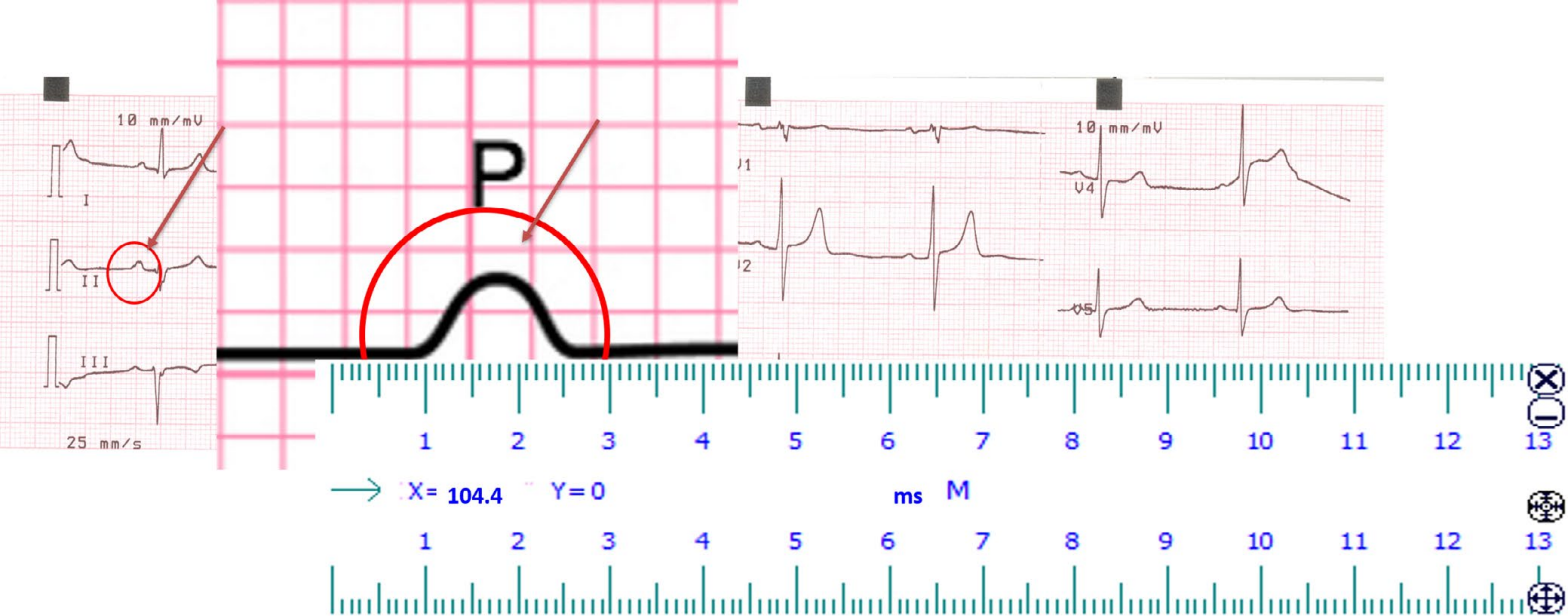
Measurement of P‐wave duration from computer screen using caliper

Twelve leads surface ECG was recorded for all patients at the study entry with a paper speed of 25 mm/s and signal size of 10 mm/mv. ECGs were scanned using a high‐resolution scanner, transferred into computer memory, magnified and P‐wave duration was manually measured from the commencement to the end of the P wave, using electronic caliper, on a high‐resolution computer screen. P‐wave start and end points were defined at the junction between P‐wave deflection and the isoelectric line. To achieve greater precision in measuring P‐wave dispersion, we measured simultaneous digital recording of all 12 ECG leads and on computer screen with the high zoom capabilities, Figure 1. P‐wave dispersion is defined as the difference between maximum and minimum P‐wave durations measured at all ECG derived leads (Acar et al., 2009; Badran et al., 2019; Liu et al., 1998; Magnani et al., 2010; Pala et al., 2010; Rein et al., 2003; Sanders et al., 2003; Tosun et al., 2018). Intraobserver and interobserver coefficients of variation were found to be 4.1% and 4.4% for PWD, respectively (Badran et al., 2019; Pala et al., 2010; Tosun et al., 2018

### 2.4 Conventional echocardiography

Echocardiographic examination was performed in the left lateral decubitus in the apical 2 & 4 chamber, parasternal long, short axis views using standard transducer positions. Esaote Mylab Gold 30 ultrasound system (Esaote S.p.A, Florence, Italy) equipped with a multi‐frequency 2.5–3.5 MHz phased‐array transducer was utilized. LV end diastolic (EDD), end systolic diameters (ESD), septum (ST), posterior wall thickness (PWT), ejection fraction (EF%), left atrial (LA), diameter, and volume (LAV) were measured in accordance with the recommendations of the American Society of Echocardiography (Maron et al., 2003).

Color flow and continuous‐wave Doppler were used to define resting LV outflow tract gradient (LVOT) and to estimate pulmonary artery pressure (PAP) from tricuspid regurgitation velocity (Bernolli equation). Peak early (E) and late (A) trans‐mitral filling velocities were measured from Doppler mitral inflow. Longitudinal peak systolic (S'), early diastolic (E'), and atrial diastolic (A') velocities as well as isovolumetric relaxation time (IRT) were obtained by placing a pulsed wave tissue Doppler (TDI) sample volume at the lateral mitral annulus in the apical four‐chamber view. E/E' was derived from the ratio between E of mitral inflow velocity and E' of annular velocity using TDI.

### 2.5 Analysis of LA deformation

Border tracking of the LA was manually traced from the digitized 2D video clips recorded during breath holding and with good quality ECG signals, which were acquired and stored for off‐line analysis using X‐Strain software with a frame rate between 40 and 80 fps. The “Zoom/RES” feature on the echocardiographic machine was used to improve the accuracy of atrial measurements. A circular region of interest was traced on the LA endocardial cavity interface in apical four‐chamber view at end diastole (LA minimum cavity area) using a point‐and‐click approach. Time–volume curves were extracted from LA wall tracking that provided automatically indexed maximum and minimum LA volume and left atrium ejection force (LAEF) (Cameli et al., 2016).

Definition of the LA endocardial border enabled the system to calculate regional longitudinal deformation of the LA walls. Peak systolic strain (ε_sys_) and LA systolic SR (SR_sys_) were measured as a positive curve at LV systole (representing reservoir function), early diastole (SR_e_) (representing conduit function), and late diastole (SR_a_) (representing contractile function). Image processing algorithm automatically subdivides the atrial wall into 12 segments distributed in septum and lateral and posterior LA wall–“roof.” The graphs for each segment were displayed and averaged to calculate global LA functions.

To estimate LA mechanical dyssynchrony, the index of myocardial systolic activation was calculated from regional strain rate curves for each segment, as time from the beginning of P wave of ECG to the peak of SR_a_ wave (TTP). LA electromechanical delay was measured as the difference between TTP (d‐TTP) in 12 LA segments (difference between the longest and shortest cycle). LA mechanical dyssynchrony was defined as the standard deviation of the time‐to‐peak SR_a_ (TTP‐SD).

### 2.6 Inter‐ and Intraobserver variability

Two independent observers performed two separate quantitative εsys and SR analyses of LA images blindly in 35 participants. Interobserver and intraobserver agreement for εsys data were LA εsys, *r* = .89 and .95, TTP, *r* = .89 and .95; and SR_sys_, interobserver *r* = .88, intraobserver *r* = .92. Both inter‐ and intraobserver agreements were lower for diastolic SR. For SR_e_: *r* = .84 and .87, respectively, and for SR_a_: *r* = .82 and .85, respectively.

### 2.7 Statistical analyses

Data were presented as numbers (%) or as mean and standard deviation values. The distribution of qualitative variables was analyzed by chi‐square test or Fisher's exact test. Quantitative variables were correlated by the use of Pearson's correlation coefficient “*r*”. All tests were two‐tailed, and *p*‐value <.05 was considered statistically significant. Receiver operating characteristic (ROC) curve analysis was performed to select optimal cutoff values of LA deformation measurements. The analysis was performed by the IBM SPSS statistics software package.

## 3 RESULTS

### 3.1 Clinical characteristics of the study population

Demographic and clinical characteristics of the study population were depicted in Table 1. There was no significant difference between patients and control subjects with respect to age, gender, BSA, HR, SBP, or DBP. HCM patients were predominantly nonobstructive 61 (77.2%) with LVOT PG < 30 mmHg, 59 (75%) had asymmetric left ventricular hypertrophy (LVH), 61 (77.2%) patient were symptomatic, and 23 (29%) were NYHA class III and IV.

**TABLE 1:** Clinical and electrocardiographic characteristics

Clinical variable	HCM (*N* = 79)	Control (*N* = 25)	p‐value	ECG Variable	HCM (*N* = 79)	Control (*N* = 25)	*p*‐value
Age (years)	43.7 ± 13.7	42.2 ± 16.9	0.762	HR (b/min)	73 ± 16.4	81.4 ± 12.5	.009
Male (%) Female (%)	53 (67.1%) 26 (32.9%)	17 (68%) 8 (32%)	0.887 0.989	PW amplitude (mv)	1.49 ± 0.52	1.32 ± 0.21	.805
BSA (m^2^)	1.82 ± 0.27	1.71 ± 0.51	0.32	PW duration (ms)	92.4 ± 14.2	65.5 ± 9.6	.000
HR (b/min)	75.9 ± 24	78.4 ± 12	0.502	Pmax (ms)	114.2 ± 17	79 ± 9.9	.000
SBP (mmHg)	128 ± 19	122 ± 8.2	0.17	Pmin (ms)	74.7 ± 16.2	52.8 ± 7.23	.000
DBP (mmHg)	80 ± 11.3	76.2 ± 8.4	0.12	PWD (ms)	42.5 ± 15.4	26.4 ± 9.8	.008
Familial	26 (32.9%)			PTF (ms.mv)	66.2 ± 48.9	25.2 ± 8.7	.0001
NYHA							
I	18 (22.8%)	25 (100%)	0.001	PR interval (ms)	164.3 ± 71.3	133.7 ± 14.9	.001
II	38 (48.1%)						
III	21 (26.6%)						
IV	2 (2.5%)						
LVOTO (%)	18 (22.8%)	0	0	QRS duration	87.9 ± 18.2	79 ± 16	.01
LVH							
Asymmetric	59 (74.7%)	0	0	QTcd	58.6 ± 14	30.3 ± 14	.001
Symmetric	20 (25.3%)						
Medications							
B blockers	66 (83.5%)	0	0	Voltage criteria	16 (20.3%)	0	.01
Ca blockers	15 (18.9%)			Repolarization abnormalities	22 (27.8%)	0	.001
Amoidaron	3 (3.8%)						

Abbreviations: BSA, body surface area, HR, heart rate, SBP, systolic blood pressure, DBP, diastolic blood pressure, PP, pulse pressure PW, P wave; PWD, P‐wave dispersion; PTF, P‐wave terminal force; QTcd, dispersion of corrected QT interval.

### 3.2 Changes in surface ECG in the study population

Almost all electrocardiographic variables showed significant difference in comparison with healthy individuals. P‐wave duration, PWD, and P terminal force were significantly higher in HCM patient (92.4 ± 14.2, 42.5 ± 15.4, 66.2 ± 48.9) versus controls (65.5 ± 9.6, 26.2 ± 8.9, 25.2 ± 8.76) *p* < .0001, <.008, <.0001, respectively. PTF was verified in 58 (73.4%) of HCM patients versus 16 (64%), in control group *p* < .01. Also PR interval was significantly prolonged in HCM patients compared with the control group (164.3 ± 71.3 vs. 133.7 ± 14.9 ms), *p* < .001. QT intervals & QT dispersion (*p* < .0001) and QRS duration (*p* < .01) were significantly prolonged in comparison with control group. Voltage criteria for LVH and repolarization abnormalities were significantly prevalent in HCM patients (*p* < .001). On the contrary, the prevalence of LBBB in HCM did not reach significant difference when compared with healthy individuals.

### 3.3 Conventional echocardiographic analysis

LA diameter, LA volume, LA volume index, fractional shortening, ejection fraction, MWT, septum, LVPW, LV mass (LVM), LVMI, DT, and E/E' were significantly greater (*p* < .0001), whereas ESD, EDD, mitral E, mitral A velocities (*p* < .0001), and mitral E/A (*p* < .02) were significantly lower in HCM group compared to control group. Twenty (25.3%) patients had moderate to severe mitral regurgitation (Table 2).

**TABLE 2:** Echocardiographic findings in HCM and control

	HCM (*n* = 79)	Control (*n* = 25)	*p*‐value
LAD (mm)	39.4 ± 8.2	27.2 ± 6.2	.000
LAV (ml)	68.1 ± 29.6	23.4 ± 11.5	.000
LAVI (ml/m^2^)	35.2 ± 14.8	15.5 ± 5.6	.000
ESD (mm)	21.8 ± 6.65	28.9 ± 5.4	.000
EDD (mm)	37.6 ± 8.46	45.6 ± 8.2	.000
FS%	42.3 ± 10.3	34.8 ± 8.8	.001
EF%	71.9 ± 11.6	64.8 ± 7.2	.000
PAP (mmHg)	33.6 ± 12.7	18.4 ± 2.8	.000
MWT (mm)	27.8 ± 7.3	8.5 ± 1.7	.000
Septum (mm)	26.6 ± 7.23	8.5 ± 1.69	.000
LVPW (mm)	16.2 ± 4.68	9 ± 1.7	.000
LVM (gm)	455 ± 186	163 ± 87	.000
LVMI (gm/m^2^)	237 ± 102	102 ± 31	.000
LVOT gradient	28.1 ± 40.2	6.1 ± 3.2	.000
Mitral E (cm/s)	76.4 ± 0.33	80.7 ± 14.9	.000
Mitral A (cm/s)	1.39 ± 6.35	51.7 ± 21	.000
No/trivial MR	28 (35.4%)	25 (100%)	.000
Mild‐Severe MR	41 (64.6%)	0	.000
mitral E/A	1.25 ± 0.54	1.46 ± 0.31	.019
DT (ms)	210 ± 84	171 ± 32	.001
E/E'	11.33 ± 5.9	4.79 ± 0.61	.000

Abbreviations: LAD, left atrium; LAV, left atrial volume; LAVI, left atrial volume index; ESD, end systolic dimension; EDD, end diastolic dimension; FS, fractional shortening; EF, ejection fraction, PAP, pulmonary artery pressure, MWT, maximal wall thickness, LVPW, left ventricular posterior wall, LVMI, left ventricular mass index, LVOT, left ventricular outflow tract; E:early mitral inflow velocity; A:atrial mitral inflow velocity, DT, deceleration time, Ea, mitral annulus early diastolic velocity.

### 3.4 LA mechanics in study population

**FIGURE 2:**
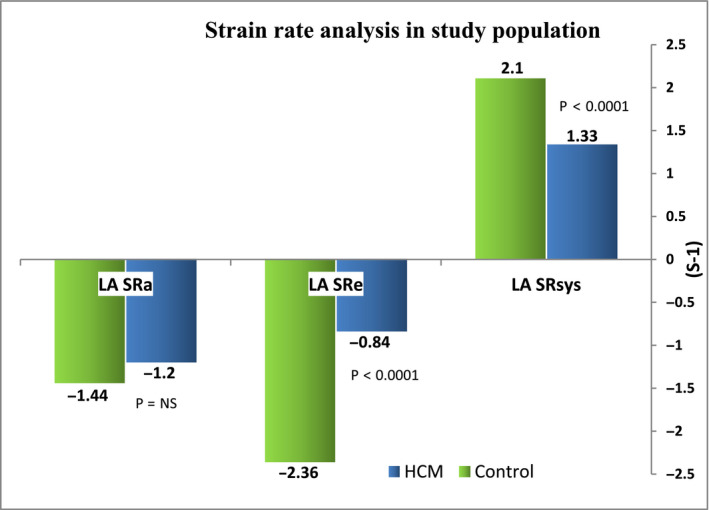
Strain rate analysis in study population

**FIGURE 3:**
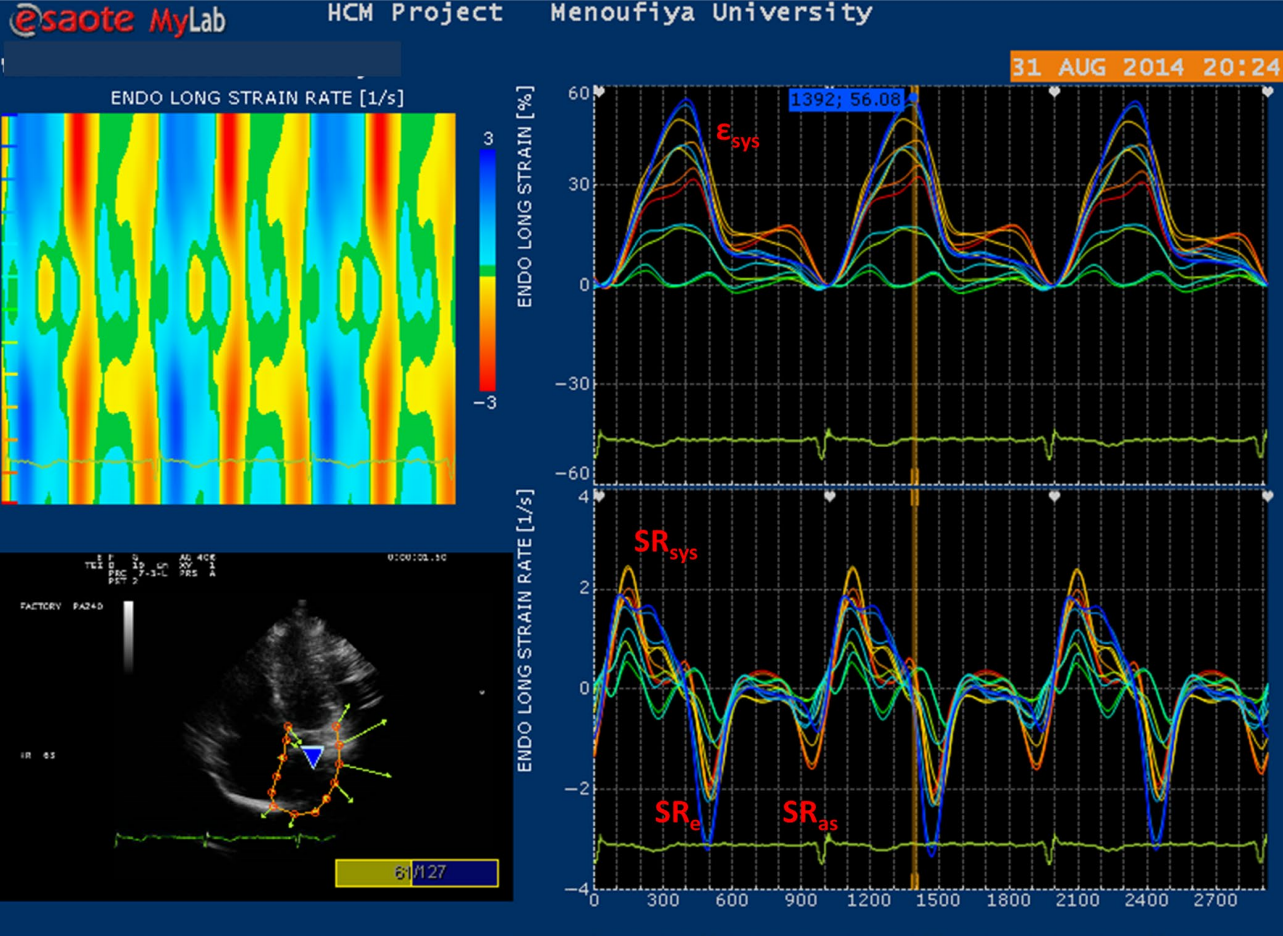
Vector velocity imaging (VVI) of left atrium in HCM patient. LA εsys and SR_sys_ measure LA reservoir function, SR_e_ measures LA conduit function and SR_a_ measures LA contractile function

LAε_sys,_ and LA SR_sys_ that reflecting LA reservoir function were significantly reduced among HCM group 24.5 ± 14.4% and 1.33 ± 0.64 s^−1^ when compared to control group, 44.4 ± 19.5% and 2.1 ± 0.55 s^−1^, *p* < .0001, respectively. Similarly, LA conduit function as denoted to LA SR_e_ was remarkably depressed in HCM group (−0.84 ± 0.47 s^−1^) compared to control group (−2.36 ± 0.87 s^−1^) *p* < .0001. No significant difference in LA contractile function (SR_a_) was observed between the two groups. LA segmental delay and dispersion of electromechanical activation were distinctly higher among HCM patient than control group as indicated by LA TTP‐ d delay and LA TTP‐SD, respectively, (95.7 ± 78.1 ms, 44.9 ± 36.1 ms) versus 27.4 ± 16.4 and 14 ± 10.4), *p* < .0001), respectively (Figures 2 and 3).

### 3.5 HCM population subgroups' according to PWD

HCM population was categorized into two subgroups according to PW dispersion ≤46 or >46 ms (2 *SD* + mean value of PWD in control group). Group I, *n* = 25 included HCM patients with long PWD, and Group II, *n* = 54 included HCM patients with normal PWD.

There was no significant difference between the two groups in age (Group 1:49.4 ± 10 years, Group 2:48.3 ± 15 years; *p* > .05), in HR, SBP, DBP, NYHA functional class, and in the use of beta blockers and calcium channel blockers. Female gender was more prevalent in group I; 14 (56%) compared with 18 (33%) women in group 2; *p* < .05. Group I had higher prevalence of LVOT obstruction, 32% versus 14% in comparison with group II, *p* < .01, (Table 3).

**TABLE 3:** Conventional ECHO and Mechanical functions in study subgroups

Variable	Group I (*n* = 25)	Group II (*n* = 54)	*p*<	Variable	Group I (*n* = 25)	Group II (*n* = 54)	*p*<
Age (years)	49.37 ± 10.2	48.34 ± 15.6	.762	HR (b/min)	67.8 ± 13.92	65.2 ± 11.8	.801
Male (%) Female (%)	11 (44%) 14 (56%)	36 (66.7%) 18 (33.3%)	.043 .047	P_max_ (ms)	127.5 ± 16.4	108.8 ± 14	.001
BSA (m^2^)	1.2 ± 0.22	1.88 ± 0.29	.01	P_min_ (ms)	69 ± 17.4^*^	77.29 ± 15.1	.006
SBP (mmHg)	136.4 ± 20.2	133.68 ± 19	.674	PWD (ms)	58.5 ± 10.9^*^	31.49 ± 8.2	.0001
DBP (mmHg)	86.2 ± 13.48	84.34 ± 11.8	.865	PTF (ms.mv)	70.9 ± 52.5	60.1 ± 37.3	.001
Familial	9 (36%)	17 (31.5%)	.802	PR interval (ms)	163.2 ± 31.7	164.9 ± 83	.898
LVOTO (%)	8 (32%)	8 (14.8%)	.02	QRS duration	84.85 ± 12.5	89.33 ± 20	.866
LAD (mm)	40.86 ± 7.2	38.75 ± 8.56	.785	QTc_d_	50.19 ± 25.6	42.5 ± 18.9	.001
LAV (ml)	72.9 ± 27.8	65.91 ± 30.4	.870	Voltage criteria	8 (32%)	8 (14.8%)	.01
LAVI (ml/m^2^)	36.9 ± 13.9	34.41 ± 15.3	.721	LBBB (%)	0	7 (13%)	.11
LVH:Asymmetric Symmetric	19 (76%) 6 (24%)	39 (72.22%) 15 (27.78%)	.645	Repolarization abnormalities	9 (36%)	10 (18.5%)	.01
ESD (mm)	22.1 ± 7.49	21.73 ± 6.29	.891	septum ε_sys_ %	23.22 ± 16.9	23.65 ± 14.5	.901
EDD (mm)	38.2 ± 8.7	37.26 ± 8.43	.672	lateral ε_sys_ %	24.65 ± 17.9	25.61 ± 17.3	.892
EF%	73.3 ± 11.2	71.28 ± 11.9	.894	Global ε_sys_ %	23.93 ± 15.6	24.74 ± 14	882
PAP (mmHg)	33.6 ± 12.7	36.12 ± 15.7	.764	Mean TTP (ms)	394.4 ± 15	293 ± 166.8	.950
MWT (mm)	28.5 ± 6.3	27.54 ± 7.77	.780	TTP‐d (ms)	120.4 ± 91	84.2 ± 68.8	.001
Septum (mm)	27.3 ± 6.11	26.24 ± 7.73	.832	TTP‐ SD (ms)	55.8 ± 41	39.9 ± 32.73	.001
LVPW (mm)	15.8 ± 3.43	16.36 ± 5.19	.905	Septum SR_sys_ (s^1^)	1.11 ± 0.74	1.3 ± 0.69	.734
LVM (gm)	505 ± 175.7	432.4 ± 187	.01	Lateral SR_sys_ (s^−1^)	1.36 ± 0.78	1.44 ± 0.79	.770
LVMI (gm/m^2^)	257 ± 96.7	228.2 ± 104	.01	Global SR_sys_(s^1^)	1.24 ± 0.67	1.37 ± 0.63	.801
LVOT PG	40.4 ± 48.6	22.14 ± 34.5	.001	septumSR_e_ (s^−1^)	−0.75 ± 0.6	−0.79 ± 0.55	.786
MitralE (cm/s)	0.89 ± 0.32	0.69 ± 0.31	.123	lateral SR_e_ (s^−1^)	−1.03 ± 0.59	−0.87 ± 0.49	.125
MitralA (cm/s)	0.73 ± 0.34	0.62 ± 0.25	.145	Global SR_e_ (s^−1^)	−0.89 ± 0.53	−0.83 ± 0.45	.864
mitral E/A	1.33 ± 0.5	1.21 ± 0.57	.210	septum SR_a_ (s^−1^)	−1.17 ± 0.86	−1.18 ± 0.64	.652
DT (ms)	204 ± 69.3	212.9 ± 91.4	0.854	lateral SR_a_ (s^−1^)	−1.14 ± 0.73	−1.25 ± 1.12	.156
E/E'	13.7 ± 5.9	10 ± 5.6	.01	Global SR_a_ (s^−1^)	−1.15 ± 0.72	−1.22 ± 0.77	.131
MR No/trivial Mild Moderate Severe	3 (12%) 14 (56%) 8 (32%) 0	25 (46.3%) 17 (31.48%) 8 (14.82%) 4 (7.4%)	.001	Medications B blockers Ca blockers Amoidaron	21 (84%) 5 (20%) 1 (4%)	45 (86.5%) 11 (21.2%) 2 (3.8%)	0

Abbreviations: LAD, left atrium; LAV, left atrial volume; LAVI, left atrial volume index; ESD, end systolic dimension; EDD, end diastolic dimension; FS, fractional shortening; EF, ejection fraction, PAP, pulmonary artery pressure, MWT, maximal wall thickness, LVPW, left ventricular posterior wall, LVMI, left ventricular mass index, LVOT, left ventricular outflow tract; E:early mitral inflow velocity; A:atrial mitral inflow velocity, DT, deceleration time, Ea, mitral annulus early diastolic velocity. εsys, peak systolic strain, TTP, time‐to‐peak strain, TTP‐SD, standard deviation of time‐to‐peak strain, SR_sys_, peak systolic strain rate, SR_e_, early diastolic strain rate, SR_a_, atrial diastolic strain rate.

P‐wave dispersion was highly significant in discriminating between the groups. Patients with increased PWD scored higher P max (*p* < .001) and lower P min (*p* < .0001). Moreover, Group I revealed higher PTF (*p* < .001), RR interval values, *p* < .01, QTc intervals, and QTc dispersion, (50.2 ± 25 vs. 42.5 ± 19 ms; *p* < .001) than group II with normal PWD, respectively. Voltage criteria for LVH and repolarization abnormalities were more prevalent in group I compared with group II, *p* < .01, while QRS duration did not differ between them. Septal thickness, PWT, EF, LA diameter, and LAVI did not differ between groups. However, patients with PWD had greater LVMI (257.6 ± 96.7 vs. 228.2 ± 104.03, *p* < .02), higher LVOT gradient (40.4 ± 48 versus 22 ± 34, *p* < .01) and elevated E/E' ratio (13.7 ± 5.9 vs. 10 ± 5.6; *p* < .01), and more prevalence of mitral regurgitation, 88% versus 53% *p* < .001 compared to those with normal P‐wave dispersion (Table 3).

**FIGURE 4:**
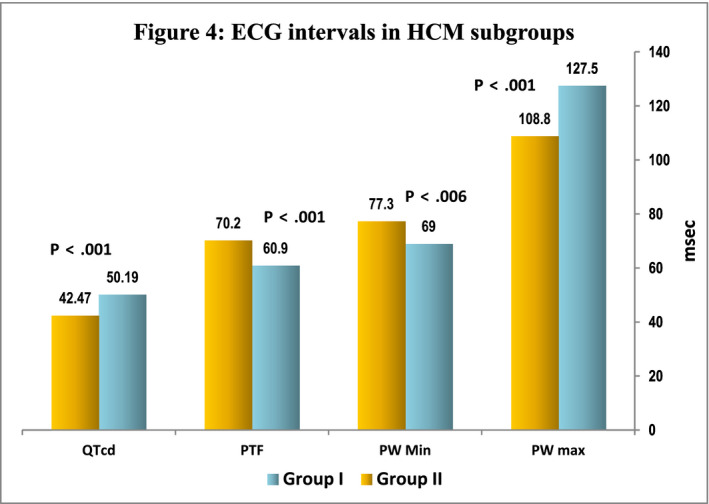
ECG intervals in HCM subgroups

Furthermore, comparing LA mechanics between HCM patients with prolonged PWD and those with normal PW dispersion, there was no significant difference in reservoir, conduit, or contractile function as measured by εsys, SR_sys_, SR_e_, and SR_a,_ respectively, *p* > .05. Only LA electromechanical activation was significantly delayed in HCM with prolonged PW dispersion, and LA TTP‐d and TTP‐SD were significantly prolonged in PW dispersion group (120.4 ± 91.9 vs. 84.2 ± 68.8, *p* < .001) and (55.8 ± 41 vs. 39.9 ± 32.3, *p* < .01) compared with patients with normal PW dispersion (Figure 4).

### 3.6 Correlates of ECG variables in patient population (table 4)

**FIGURE 5:**
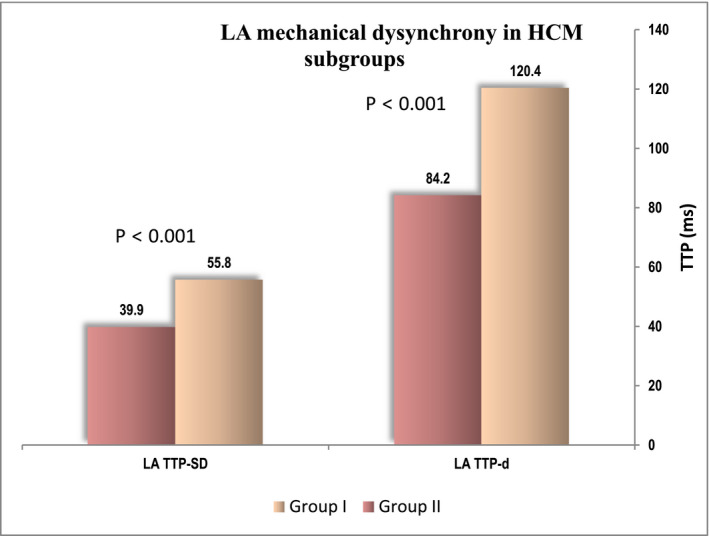
LA mechanical dysynchrony in HCM subgroups

**FIGURE 6:**
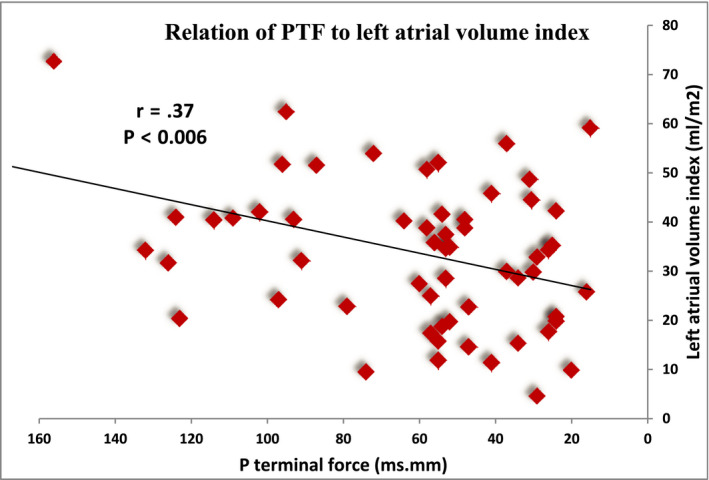
Relation of PTF to left atrial volume index

Taking the HCM patients altogether, Pearson's correlation coefficient was utilized to study the interaction between ECG variables functional status and LA structure and function. No significant correlation was noted between NYHA class and PWD (*r* = .11, *p* < .36), PTF (*r* = .14, *p* < .32), or PR interval (*r* = .13, *p* < .25), respectively. P terminal force was inversely correlated with LA volume and left atrial volume index (Figure 3) (*p* ˂ .004, *p* ˂ .006) and to a lesser extent to LA diameter and MR severity (*p* ˂ .02, *p* ˂ .04). There was a more strong direct correlation between PR interval and EDD (*p* ˂ .003) than ESD (*p* ˂ .05). However, no significant correlations existed between P‐wave dispersion or other ECG variables and LA and LV mechanics parameters (Figures 5 and 6). So ECG variables were related to atrial size and structure but not related to functional parameters as derived from 2D strain imaging. Meanwhile, no significant correlation was detected between P‐wave indices and QRS duration or QTc intervals.

## 4 DISCUSSION

P‐wave related entities on surface ECG are important electrical parameters that should be assessed in patients with hypertrophic cardiomyopathy. In the current study, PWD was associated with more severe phenotype, higher E/E', more severe mitral regurgitation, and LA electromechanical dyssynchrony. In HCM, PWD is associated with higher PTF and QTc interval prolongation and dispersion. PTF was correlated solely to atrial enlargement and structural remodeling. PWD could be used to discriminate HCM patients with atrial electromechanical heterogeneities that might progress to atrial fibrillation (AF).

Patients with HCM are more likely to develop AF when compared with healthy individuals (Gersh et al., 2011; Maron et al., 2002). Occurrence of AF initiate further clinical deterioration, thromboembolic complications, and increase mortality in this population (Olivotto et al., 2001). Therefore, early identification of HCM patients who are at a higher risk to develop AF is critical to prevent its harmful consequences.

Numerous studies have focused on predictors of AF in patients with HCM. Advancing age, functional status, LA enlargement, P‐wave duration, and extent of LV fibrosis have been suggested to predict the propensity to develop AF in these patients (Cecchi et al., 1997; Olivotto, et al., 2001; Olivotto et al., 2001; Yamaji et al., 2001). Several investigators studied the changes in P‐wave related entities for instance, coronary artery diseases, coronary artery bypass graft, hypertension, heart failure, and paroxysmal AF (Dilaveris et al., 1999; Frost et al., 1996; Yamada et al., 2000). Recently, PWD has been proposed to be a useful index for identifying AF.

Meanwhile, there are well‐established electrophysiologic characteristics that predispose to AF development including delayed intra‐ and interatrial electrical conduction time, an increased atrial electrocardiographic fragmentation, and an increased dispersion of atrial refractoriness (Abhayaratna et al., 2008; Suga, 1974; Tuluce et al., 2014). These properties reflected in P‐wave morphology and duration on the 12‐lead ECG. Electromechanical inhomogeneities of atrial myocardium in HCM population are usually associated with generation of unidirectional block of premature impulses, hence trigger atrial reentry (Kose et al., 2003).

In our study, we tried to find the relation between ECG derived atrial electrical parameters like PWD, PTF, and atrial mechanical function using VVI that precisely measure atrial phasic functions and electromechanical delay.

In the present study, 32% of HCM patients had significantly increased dispersion of P‐wave duration; both maximum and minimum P‐wave durations were significantly different from those with no dispersion. Higher PWD on the surface ECG probably thought to reflect the heterogeneity of mechanical and electrophysiologic properties of atrial myocardium.

In our study, LA diameter and LAVI were correlated directly with PTF that reflects particularly atrial remodeling, while PWD was associated more with ventricular structure and the severity of HCM phenotype. Indeed, patients with PWD showed increased LVMI, higher LVOT gradient, more severe mitral regurgitation, and the resulting increased LV filling pressure as denoted by E/E'. The most widespread cause of increased LA size and propensity to AF in patients with HCM is the presence of obstruction concomitant with mitral regurgitation (Tuluce et al., 2015).

Furthermore, atrial electromechanical dyssynchrony was significantly manifest in HCM with PWD but was not correlated with PTF. This can be verified by the fact that the extremely anisotropic properties of the atrial myocardium due to microarchitectural changes in HCM might be more crucial in the genesis of AF paroxysms than cavity size (Lavergne et al., 1986; Misier et al., 1992). Fananapazir et al. (1989) investigated 155 HCM patients using electrophysiologic study, and they reported that 66% had prolonged sinoatrial conduction time. The presence of prolonged and fractionated atrial electrograms revealed a strong association with paroxysmal AF induction.

The importance of prolonged PWD in distinguishing HCM patients who are prone to AF risk from those without was previously investigated; however, the value of PWD in predicting future development of AF in a follow‐up study has not been determined yet. Kose et al. (2003) categorized patients with HCM into two groups: group I with and group II without paroxysmal AF attacks, and compared their PWD to identify the value of PWD in prediction of AF. In their study, they demonstrated a value of 46 ms as a cutoff point of PWD that differentiates patients with previous PAF attacks with 76% sensitivity and 82% specificity.

Another study conducted to estimate predictors of AF in HCM included 27 patients with HCM with a previous history of documented AF attack and 53 patients who had no such history (Ozdemir et al., 2004). This study demonstrated a PWD value of >52.5 ms differentiate patients from controls with a sensitivity of 96%, a specificity of 91%, and a positive predictive accuracy of 84%.

In our study, we used comparable PWD cutoff value derived from 2SD of mean value of PWD in healthy individuals. Our study had a similar number of patients compared to their patient number; however, our study not designed as a follow‐up study.

Moreover, our study HCM patients with PWD had no larger LA size but higher LV end diastolic pressure as estimated by E/E' and more electromechanical delay. Our study confirm the findings by Tuluce et al. (2015), and they illustrated that electrical LA remodeling and not structural impairment has the main role in promoting AF.

ECG would be a straightforward, inexpensive, readily accessible, and noninvasive means to diagnose atrial electromechanical dyssynchrony if reliable criteria are available. PWD can be used as bed side prognostic test that able to discriminate HCM patients with atrial electrical heterogeneity at higher risk to develop AF. The criterion used in the present study is one of the most frequently used clinically which is PWD. Increased PWD of left atrial electrical properties is thought to reflect the heterogeneity of atrial electromechanical properties and electrophysiologic parameter that reflect prolongation of intra‐atrial and interatrial conduction time and the inhomogeneous propagation of sinus impulses. This confirms the findings of other studies (Flaker et al., 1995; Henry et al., 1976; Ozdemir et al., 2004; Turitto et al., 1998).

### 4.1 Clinical implication

The phenotypic variability of HCM is not only limited to variability of severity and extent of myocardial hypertrophy, presence or absence of obstruction, presence of absence of mitral regurgitation but also rather includes a set of functional manifestations and the electrophysiologic properties (Maron et al., 2011; Moon & McKenna, 2012; Soler et al., 2018). It is important to look for prognostic markers that identify patients more prone to adverse cardiovascular events. This study shows considerable importance of most of ECG parameters including P‐wave duration, P‐wave dispersion, PTF, and PR intervals, as part of electrical characteristics of the disease, and adds to clinical evaluation and risk stratification of HCM population.

## 5 STUDY LIMITATIONS

There are some inevitable limitations to our work. First, the sample size of our HCM population is relatively small. Second, patient follow‐up was lacking to detect the occurrence of AF in patients with electromechanical dyssynchony. However, the less common AF development and the very incidental progress to AF in HCM patients, render a longitudinal study be unrealistic for most clinical research. P‐wave measurements from surface ECG need precision; however, it is often cumbersome in clinical practice, especially with the modest quality of P‐wave inscriptions on the ECG, so it is expected to reduce the clinical value of these observations. Further prospective clinical studies are needed to validate the role of PWD in predicting AF in patients with HCM.

## 6 CONCLUSION

We concluded that PWD is associated with more severe HCM phenotype and left atrial electromechanical delay, while PTF is linked to atrial remodeling. The ECG parameter, if properly ascertained, emerges to be a practical criterion of atrial electromechanical delay.

PWD is revealed as a valuable parameter of easy measurement that signifies a greater tendency to the development of supraventricular arrhythmias, particularly AF. P‐wave related entities may be considered as ECG‐based marker of LA remodeling and predictors of increased risk of AF occurrence in HCM patients. If echocardiography is not available, ECG can be helpful for estimation and quantification of LA size and electromechanical properties.

## Conflict of interest

All authors declare that there is No conflict of interest.

7

**TABLE 4:** Relationship of ECG variables to cardiac mechanics in HCM

		PWD	PTF	PR interval			PWD	PTF	PR interval
Age (years)	*r*	−.029	.081	−.106	LA Global εsys %	*r*	.053	.175	.034
	*p*	.803	.541	.353		*p*	.641	.184	.767
Functional class	*r*	.113	.142	.131	LA Global SR_sys_	*r*	−.015	.189	−.043
	*p*	.360	.325	.250		*p*	.898	.152	.705
LA diameter (mm)	*r*	.004	.335	−.023	LA Global SR_e_	*r*	−.061	−.184	−.076
	*p*	.974	.010	.843		*p*	.594	.164	.507
LA volume (ml)	*r*	.126	.378	−.073	LA Global SR_a_	*r*	−.012	−.111	−.111
	*p*	.267	.003	.520		*p*	.917	.402	.332
LA volume index (ml/m^2^)	*r*	.128	.365	−.140	LA TTP‐SD	*r*	−.091	−.034	.172
	*p*	.261	.005	.220		*p*	.423	.799	.129
DT (ms)	*r*	−.007	.123	.008	LA TTP‐d	*r*	−.078	−.018	.183
	*p*	.949	.351	.943		*p*	.495	.892	.107
E/E'	*r*	.099	.061	−.104	LV global εsys %	*r*	−.021	−.102	−.101
	*p*	.388	.648	.365		*p*	.856	.445	.379
LA Ejection Fraction	*r*	.026	.102	.047	LV Global SR_sys_	*r*	−.099	−.102	−.122
	*p*	.821	.441	.682		*p*	.388	.446	.288
ESD (mm)	*r*	.078	−.122	.230	LV Global SR_e_	*r*	.069	.123	.185
	*p*	.498	.358	.043		*p*	.550	.357	.104
EDD (mm)	*r*	.134	−.065	.343	LV Global SR_a_	*r*	.049	.186	−.001
	*p*	.244	.623	.002		*p*	.673	.162	.992
EF%	*r*	.041	.105	.042	MR severity	*r*	.194	−.275	−.199
	*p*	.720	.429	.712		*p*	.086	.033	.079
QRS	*r*	−.014	−.210	−.094	OTc	*r*	−.047	−.026	−.016
	*p*	.903	.111	.409		*p*	.684	.846	.889
